# Correlation Analysis between Hemoglobin and C-Reactive Protein in Patients Admitted to an Emergency Unit

**DOI:** 10.3390/jcm10225411

**Published:** 2021-11-19

**Authors:** Miguel A. Santos-Silva, Nuno Sousa, João Carlos Sousa

**Affiliations:** 1 Life and Health Sciences Research Institute (ICVS), School of Medicine, University of Minho, Campus Gualtar, 4750-057 Braga, Portugal; id8999@alunos.uminho.pt (M.A.S.-S.); njcsousa@med.uminho.pt (N.S.); 2 Life and Health Sciences Research Institute (ICVS), School of Health Sciences, University of Minho, Campus Gualtar, 4805-017 Braga, Portugal; 3 Enlightenment, Lda, 4750-057 Braga, Portugal; 4 Clinical Academic Center-Braga (2CA), 4710-243 Braga, Portugal

**Keywords:** hemoglobin, C-reactive protein, emergency, correlation

## Abstract

Anemia and inflammation are common clinical conditions in emergency departments. This study explored a cohort of patients admitted to the emergency department with a particular interest in determining the frequency of anemia and inflammation and the association between hemoglobin (Hb) and C-reactive protein (CRP) concentrations. The study included 125 patients categorized according to their demographic (gender and age) and clinical condition (Hb and CRP concentrations, pathological background, and diagnostic). We found that anemia and inflammation were simultaneously present in 36.0% of the cohort, reaching 67.0% in patients that were subsequently hospitalized. The Hb level was significantly lower in patients with elevated concentration of CRP when compared to individuals with normal CRP levels (11.58 ± 2.23 vs. 13.25 ± 1.80, *p* = 0.001); furthermore, we found a significantly negative correlation between Hb concentration and the CRP level (rs = −0.42, *p* < 0.001). The linear regression model applied to the cohort showed that CRP levels explain 15% of Hb variations. The sensitivity of the CRP/Hb ratio (cut-off = 1.32) as a predictor of hospitalization was 80.0%, with a specificity of 68.4% for all patients. These findings confirmed the prevalence of anemia and inflammation and identified a moderate but significant association between Hb and serum CRP in a heterogeneous group of patients admitted to the emergency department.

## 1. Introduction

Admissions to emergency departments (EDs) comprise a very heterogeneous population, from patient demographics to the type of symptoms and conditions (chronic or acute) that require immediate care [1]. Although many studies are conducted for specific pathologies with highly isolated groups of individuals, many of the patients admitted to EDs present multiple symptoms and comorbidities, often associated with chronic pathologies that lack characterization [2]. The association between the clinical history, symptoms, and data from exams such as blood laboratory analysis is relevant to understanding the patient’s health problem [3]. Furthermore, the information retrieved from blood laboratory analysis influences 70% of medical decisions [4]. Thus, this work focused on addressing whether there is an association between two of the most common blood metabolites evaluated in the context of ED admissions, i.e., hemoglobin (Hb) and C-reactive protein (CRP), with the diagnostic made on a patient’s admission.

According to the World Health Organization (WHO), the prevalence of anemia varies between 22.9% and 26.7% (95% CI), affecting almost 1.62 billion people [5]. Typically, anemia encompasses low levels of circulating red blood cells (RBCs) or Hb concentration and is defined with Hb threshold values of <13 g/dL and <12 g/dL for men and women, respectively [5]. Laboratory characterization of anemia is accomplished either by a morphologic or kinetic approach. The morphological approach categorizes anemia according to RBC mean corpuscular volume (MCV), namely microcytic (MCV < 80 fL), normocytic (80–100 fL), and macrocytic (>100 fL) anemias. The kinetic approach differentiates anemias caused by the increased loss of RBCs (due to chronic or acute hemorrhages), increased destruction of RBCs (caused by acquired or inherited factors), and decreased production of RBCs (due, for example, to the lack of essential vitamins or hormones, or when the patient undergoes chemotherapy or exposure to radiation) [6].

The most prevalent cause of anemia is iron deficiency (IDA), followed by anemia of chronic disease (ACD) [7]. ACD, also named anemia of inflammation, has a high prevalence, particularly in the elderly (70%), due to the continuous increase of chronic diseases. ACD is mainly caused by infections (chronic or acute), cancer, autoimmune disorders, chronic rejection after solid-organ transplantation, and chronic kidney disease and inflammation [7]. Recently, congestive heart failure, chronic pulmonary disease, and obesity have also been associated with ACD [8]. Briefly, these pathologies stimulate the production of proinflammatory cytokines that enable iron sequestration and cause shortened RBC survival [9]. In the case of emergency departments specifically, existing studies reported significant variations of Hb concentration, with an average prevalence of 44% [10] anemic individuals, independent of age and gender. Notably, this value increases in the elderly cohort (+70 years), and it is higher in males than females [11].

CRP is an acute-phase protein and pattern-recognition marker of systemic inflammation. CRP is frequently measured in emergency settings and synthesized in the liver in response to cytokines or the tumor necrosis factor produced during infections or inflammatory mechanisms [12,13]. The physiological behavior of CRP involves a 4–6 h increase after inflammation initiates, doubling its value every 8 h with a peak at 36–50 h, ending with a sharp decrease once the inflammation is resolved [14]. However, increased CRP concentrations over time, rather than spikes, are intrinsically related to several chronic pathologies, namely cardiovascular diseases, atherosclerosis, hemorrhagic stroke, Alzheimer’s disease, and Parkinson’s disease [15].

Some studies describe the relationship between Hb and CRP. While Ziv-Baran et al. found a marginal (rs = −0.025, *p* < 0.001) association between both proteins in 16,095 healthy individuals undergoing a routine health examination, Heidari et al. described a moderate linear association (rs = −0.607, *p* = 0.001) between Hb and CRP in 73 hemodialysis patients [16]. Although these studies are not comparable as they focus on substantially different cohorts, the association between Hb and CRP was analogous, confirming its possible relevance for clinical diagnosis and prognosis. This aspect was mostly visible in the hemodialysis study, where the inflammatory process, as evaluated by CRP levels, reduced Hb levels linearly [17]. Additionally, it is well-known that decreasing Hb levels linked to inflammatory states highly correlate to higher mortality [18]. Monitoring Hb in patients with inflammatory conditions is, thus, of relevance for patient and treatment monitoring [19].

Nevertheless, whether Hb and CRP associate in patients admitted to the emergency department has not yet been studied, which is what we approached in this work. Additionally, we explored how this potential association would relate to patient diagnosis at admission and whether it could support the clinical decision in the context of emergency department admission.

## 2. Materials and Methods

### 2.1. Study Design and Population

This work is a retrospective observational study of Portuguese individuals who attended the emergency department of the Hospital de Braga between May and December of 2019. The hospital assigns an identification code to compile the patient’s clinical information longitudinally. The database contains demographic information (gender and age), clinical information (clinical analyses and exams results), and information regarding past and future appointments or procedures. This identification code allows for a direct and anonymous connection to each patient, according to the ethical guidelines of the Braga Hospital Ethics Committee. The study was approved by the Portuguese data protection commission, and the Braga Hospital and the University of Minho Ethics Committees (SECVS-011/2013) under the project “My Health DoIT”.

The study focused on a sample of adult patients (18 years or older) with entire clinical history, including pathological background, symptoms description, length of stay, and blood analysis. The sample collection method was random, namely on non-consecutive days and on different time intervals over the various days, to avoid repeated causes of emergency department arrivals (for instance, acute myocardial accidents, which are prevalent in the morning). The blood analysis comprised a complete blood count (CBC) and chemistry panel. Participants with incomplete information regarding clinical history were excluded. Only blood tests with complete cell counts and Hb and CRP concentrations were considered since one of the study objectives was an evaluation of the relation between Hb and CRP concentrations.

### 2.2. Data Source, Measurement, and Variables

Patients’ anonymized demographic (gender and age) and clinical (pathological background, symptoms description, length of stay, and blood analysis) data were retrieved from the Clinic Academic Center (2CA, Braga, Portugal) of the Hospital de Braga. CBC, including erythrocytes, platelets, and white blood cells count, and Hb concentration, hematocrit ratio, and red cell distribution width, were analyzed in the hospital clinical pathology laboratory using standard methods (Sysmex XE-2100, Sysmex Inc, Mundelein, IL, USA). Serum CRP was measured with traditional immunoturbidimetric assay by the ADVIA system (Siemens Healthcare Diagnostics Inc, Tarrytown, NY, USA).

Symptoms, pathological background, and primary diagnostic performed in the emergency department were merged according to the International Statistical Classification of Diseases and Related Health problems 10th revision (ICD-10). Length of stay was calculated as the difference between the time the patient was screened in the emergency department and the final hospital discharge, including exits to hospitalization, primary health care centers for follow-up by the family medicine physician, or home stays.

### 2.3. Statistical Analysis

Statistical analysis of the data grouped patients based on gender, age, primary diagnosis, and Hb and CRP concentrations. Furthermore, the most frequently diagnosed ICD-10 groups were explored as a function of age and Hb and CRP concentrations. This analysis was performed with SPSS (IBM SPSS Statistics for Mac, version 26, IBM Corp., NY, USA).

Continuous variables were evaluated for normal distribution with histograms (skewness and kurtosis) and described using the mean and standard deviation (SD). Skewed continuous variables were reported with median and interquartile range (IQR). Categorical variables were described using frequency and percentage. The association between Hb and CRP was evaluated with Spearman’s correlation coefficient. The comparison between CRP in different categorical groups was accomplished with Kruskal–Wallis and Mann–Whitney tests. Receiver operating characteristic (ROC) analysis was used to evaluate the predictive ability of both Hb and CRP for the hospitalization discharge. The area under the ROC curve (AUC) was calculated together with 95% confidence intervals and assessed accordingly: 0.5–0.6: fail, 0.6–0.7: poor, 0.7–0.8: fair, 0.8–0.9: good, and 0.9–1.0: excellent. The Youden index (maximum sensitivity and specificity) was applied to determine the best cut-off value for the significant studied parameters in ROC analysis and evaluated with sensitivity and specificity. Statistical significance was determined at *p* < 0.05.

## 3. Results

### 3.1. Participants and Clinical Condition

The study cohort (Table 1) included data of 125 individuals (50.4% males and 49.6% females) with a median age of 69 years (IQR 49–81 years). Anemia was defined according to the WHO threshold, with values of Hb of less than 12 g/dL for women and less than 13 g/dL for men. An active inflammatory process was considered when CRP was above 5 mg/L. Diagnostic groups of diseases (ICD-10) with less than 5% of cases, such as hematological, metabolic, as well as mental conditions, and conditions of the ear, skin, musculoskeletal, injury or poisoning, and external causes of morbidity and mortality, were not included for further analysis.

According to the criteria indicated above for the definition of each clinical condition, total anemia (irrespective of CRP levels) and total inflammation (irrespective of Hb levels) displayed a prevalence of 44.8% and 63.2%, respectively. Anemia was more present in males (57.1%) rather than females (42.9%) and was five times higher in patients aged 65 or older than in younger subjects (84.0% against 16.0%). Considering the diagnostic groups, the prevalence of anemia decreased from neoplasms (100%) to diseases of digestive (53.3%), genitourinary (43.5%), nervous (42.9%), infectious (41.6%), respiratory (30%), and circulatory (14.3%) systems. Only 7.2% of anemic patients were discharged to have health care at home.

Active inflammation was found in 63.2% of the cohort, balanced between gender but with approximately twice the rate in senior patients (age > 65 with 70.0%). Patients with CRP ≥ 5 mg/L were found in more than 50.0% for each diagnostic group of diseases. Respiratory diseases had 81.2% of patients with elevated CRP, followed by neoplasms (71.4%), infectious (66.6%), and cardiovascular and digestive diseases (both with 60.0%), diseases of the genitourinary system (65.0%), and diseases of the nervous system (57.1%).

When considering anemia only (no alterations in CRP levels), the prevalence was 8.8%. It affected more males (54.5%) than females (45.5%) and was nearly three times higher in patients aged 65 or older than in younger subjects (73% vs. 27%). When considering the diagnostic groups, the prevalence of anemia was higher in neoplasms (28.6%), infectious disorders (16.7%), digestive diseases (13.3%), and genitourinary diseases (13.0%) (Table 2). In our small cohort, patients with diseases of the nervous and respiratory systems were not anemic. Only 9.1% of anemic patients were hospitalized, while 18.2% were discharged to follow health care at home, and the remaining patients were signaled to be monitored at a primary health care center.

Active inflammation only (no alterations in Hb levels) was found in 24.8% of the samples, evenly distributed between age groups but with approximately 1.5 times more cases in female patients (61.3% against 38.7%). Patients with CRP ≥ 5 mg/L were found in all diagnostic groups except for neoplasms. Elevated CRP levels were detected in 41.7% of patients with infectious diseases, followed by circulatory (35.0%), genitourinary (34.8%), digestive (20.0%), and respiratory diseases (18.8%), and diseases of the nervous system (28.6%). In this cohort, patients with neoplasms did not display CRP levels above 5 mg/L. In opposition to anemia, an active inflammatory condition was more prevalent in patients with a high length of stay (>6 h) in the emergency department (54.8% against 45.2%).

Patients with both clinical conditions (anemia and active inflammatory process) represented nearly 36% of the sample population. When considering gender and age distribution, respectively, the existence of both anemia and an active inflammatory process was more prevalent in males (57.8%) and patients aged ≥65 years (86.7%). Neoplasms were the most prevalent (71.4%), followed by diseases of the respiratory system (62.5%), digestive system (40.0%), genitourinary system (30.4%), and infectious diseases and diseases of the cardiovascular system (25.0%). Interestingly, patients without any of these clinical conditions corresponded to the second most prevalent group of the cohort (30.4%); in this group, only two patients were hospitalized, and the most common diagnostic was related to diseases of the circulatory system (18.4%).

The median age of the study population also represented differences by diagnosis group: patients under the age of 65 years had a higher prevalence of diseases of the nervous system (VI, 9.6% vs. 2.7%), while the aged group (≥65 years) had a higher proportion of diseases of the genitourinary system (XIV, 20.5% vs. 15.4%).

### 3.2. Correlation between Hemoglobin and C-Reactive Protein

Given that the level of Hb was significantly lower in patients with elevated concentration of CRP when compared to the normal group (11.5 ± 2.2 vs. 13.2 ± 1.8, *p* = 0.001), we next evaluated how strongly these metabolites were correlated.

**Figure 1:**
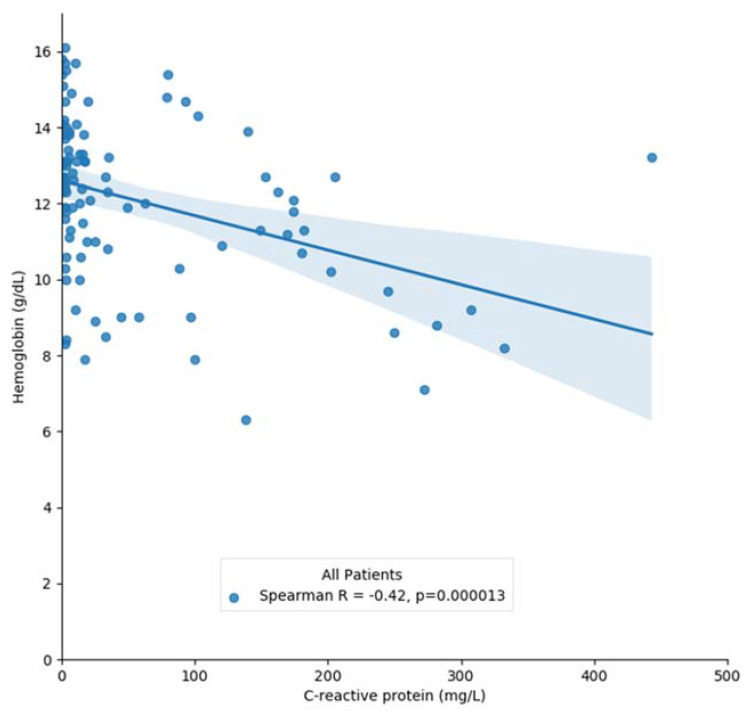
Correlation between hemoglobin and C-reactive protein concentrations in the entire cohort.

When considering the entire cohort, we found a significantly negative correlation between the Hb concentration and the CRP levels (rs = −0.42, r^2^ = 0.15, *p* < 0.001) (Figure 1). This association was maintained after adjustments for both gender and age groups. Interestingly, the correlation was stronger in males than females (rs = −0.56 vs. rs = −0.37, *p* < 0.05) and in the aged group (rs = −0.43 vs. rs = −0.30, *p* < 0.05).

In patients grouped by diagnostics, the Spearman’s correlation was significant for patients with diseases of the nervous system (rs = −0.82, *p* < 0.05) and respiratory system (rs = −0.77, *p* < 0.05). Patients with digestive system diseases displayed a correlation of –0.509 that nevertheless failed to reach statistical significance (*p* = 0.053).

When considering the different types of discharge and lengths of stay, a significant correlation between Hb and CRP levels was also verified. However, this was not confirmed when the cohort was analyzed by clinical condition. In fact, only patients with both anemia and inflammation presented a significant association between Hb and CRP, particularly in hospitalized patients and in patients diagnosed with respiratory diseases (Table 3).

### 3.3. Hemoglobin and CRP Distribution

**Figure 2:**
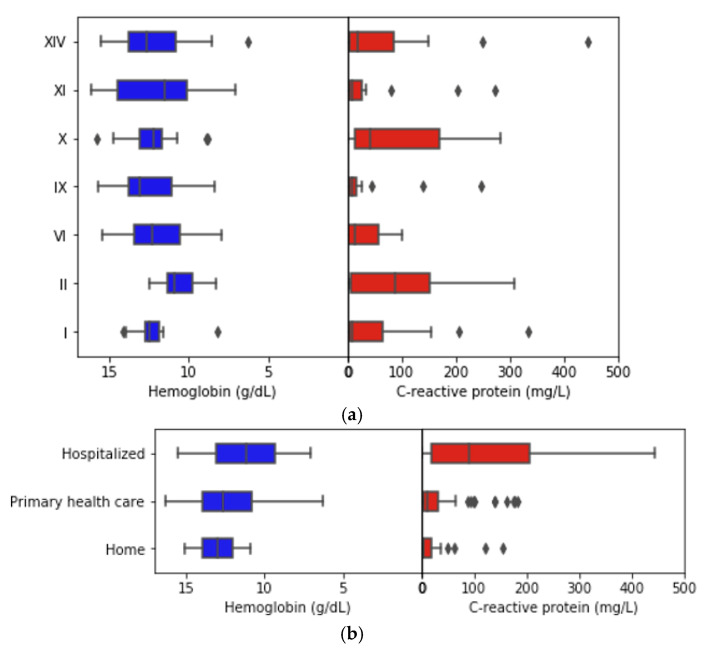
Boxplots show the distribution of hemoglobin and c-reactive protein according to diagnostics group (**a**) and type of discharge (**b**). Rectangles represent the second and third quartiles with a vertical line inside to indicate the median value. The lower and upper quartiles are shown as horizontal lines on either side of the rectangle and outliers are plotted outside of it, individually.

We next analyzed the distribution of the concentration of Hb. The study population displayed a normal distribution of Hb levels with an average of 12.2 ± 2.2 g/dL and a median value of 12.6 (10.8–13.9) g/dL. It did not differ between genders [t(113.6) = −0.839, *p* = 0.403], but varied significantly between age groups [t(123) = 4.558, *p* = 0.001]. When considering the diagnostic groups (ICD-10), the concentration of Hb was similar across pathologies, except for neoplasms [t(123) = 2.105, *p* = 0.037] with patients with oncologic conditions presenting lower Hb concentrations (Group II) (Figure 2a).

CRP concentration in the study population displayed a left-skewed distribution and a median value of 10.8 (IQR 2.8–63.7 mg/L). It was not statistically different between genders [H(1) = 0.098, *p* = 0.754] but differed between age groups (U = 1202.5, z = −3.564, *p* < 0.001). Furthermore, the median levels of CRP were only significantly different for patients with diseases of the respiratory system (U = 578,5, z = −2.171, *p* = 0.030).

Discharge groups exhibited a similar behavior regarding the comparison between the levels of Hb and CRP. Patients that were admitted for hospitalization presented lower Hb [F (2,18) = 4.338, *p* = 0016] and higher CRP levels [H(2) = 26.31, *p* = 0.001] than patients oriented for primary health care or home care. No statistical differences were observed in the levels of both blood metabolites on the two latter groups of patients (Figure 2b). Therefore, this suggests that Hb and CRP concentrations at admission might act as discharge predictors for hospitalization when a patient visits the emergency department.

Additionally, the concentration of CRP was also different according to the length of stay in the emergency department [H(1) = 5.298, *p* = 0.021], with a cut-off point of 6 h. Hb levels did not differ between the different length of stay groups [t(123) = 1.431, *p* = 0.155].

### 3.4. ROC Analysis

**Figure 3:**
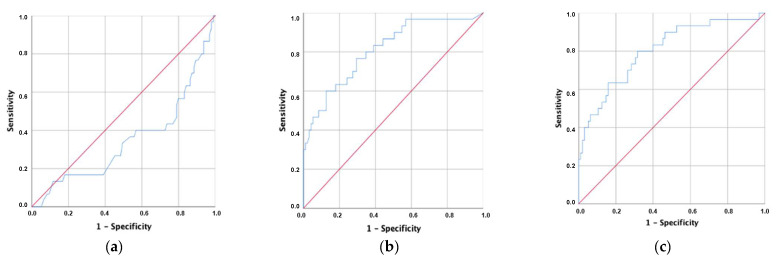
ROC curves of hemoglobin (**a**), C-reactive protein (**b**), and C-reactive protein-to-hemoglobin ratio (**c**).

Considering the significant differences in the Hb and CRP levels between discharge groups, ROC analysis was performed to evaluate the area under the curve (AUC) for the classification metrics regarding the destination of patients after emergency room discharge. The AUC was found to be statistically significant across the three analyses but decreased from CRP (0.811) to CRP/Hb (0.804) and Hb (0.347) (Figure 3).

The sensitivity of the CRP/Hb score (cut-off = 1.32) in the discharge for hospitalization was 80.0%, and the specificity was 68.4% for patients arriving at the emergency department. The analysis performed for Hb revealed that it did not have predictive value for discharge destination from the ER (AUC < 0.5) (Table 4).

## 4. Discussion

Data regarding the relationship between serum CRP and Hb concentration are limited. To the best of our knowledge, their association was not determined in heterogeneous patients admitted to emergency settings. This retrospective study performed an exploratory evaluation of patients admitted to an emergency department considering the concentration of Hb and CRP as markers of anemia and inflammation, respectively. The association between both metabolites was studied globally and sub-segmented according to their demographics, diagnostics ICD-10 classification, type of discharge, and length of stay in the emergency department.

The results of this study indicate that Hb and CRP blood concentrations strongly associate. Average Hb concentration was significantly lower in patients with a high concentration of CRP. Serum CRP negatively correlated with Hb concentration, and its relationship remained significant after adjustment for gender, age group, specific group of pathologies, discharge, and length of stay in the emergency department. Interestingly, a significant correlation between both blood parameters was verified in the 125 patients studied, and the linear regression model applied showed that the levels of CRP explained 15% of Hb variations. Considering each patient’s multitude of symptoms and pathological background, the correlation herein described is of clinical significance. Reports from studies exploring the association between these blood metabolites found differences according to the cohort type. Ziv-Baran et al. (2020) studied apparently healthy individuals undergoing a routine health examination. They found a general significant, albeit weak, relationship between these blood metabolites, namely an inverse correlation between serum CRP and Hb (r^2^ = −0.025, *p* < 0.001). Furthermore, a correlation between the blood concentration of these metabolites was found across different age groups and in males [16]. However, this study lacked detailed clinical characterization of the cohort.

In the present study, we categorized patients according to the diagnostics made on emergency department admission and conducted linear associations between the metabolites studied. Hb levels displayed a normal distribution, without significant differences across ICD groups, except for patients with active cancer (neoplasms). Furthermore, the prevalence of anemia (levels of Hb below 13 mg/dL or 12 mg/dL in males and females, respectively) in the cohort agrees with the literature [10], with an average of 40%. Additionally, and in accordance with the reported by others [20], we found that anemia is more prevalent in males and in the elderly group (>65 years). Different from Hb, CRP concentration exhibited a left-skewed distribution that was only significantly higher for aged patients and those diagnosed with diseases of the respiratory system.

Considering the type of discharge, we found that both anemia and inflammation frequencies increased from home to primary health care and hospitalization. Hospitalized patients displayed significantly different levels of both Hb and CRP, with symmetric (negative) variation (correlation). This correlation was still significant for 67% of patients with both anemia and inflammation, suggesting that the increase in CRP explains nearly 45% of the decrease in Hb. These findings follow the current knowledge regarding the impact of anemia of inflammation in determining long-term hospitalization and increased mortality outcomes [19].

The evaluation of Hb and CRP as predictors of discharge from the emergency unit to hospitalization showed relevant predictive efficiency for CRP and the CRP/Hb ratio. This test confirms the importance of the CRP concentration as a predictor of hospitalization. We suggest that the cut-off value of 1.32 for the CRP/Hb ratio (with a sensitivity of 80.0%) might be considered as a relevant biochemical index that could provide support at admission to emergency units in discriminating between patients with a need for hospitalization.

This retrospective observational study presents limitations such as the reduced sample size, which limited in-depth statistical associations between gender, age, and disease groups and clinical outcomes regarding the longitudinal behavior of blood metabolites. A collection of anemia-related biomarkers, such as ferritin and transferrin saturation, would also be relevant to differentiate patients according to the type of anemia. Correlations between studied metabolites could also be more personalized and valuable in that context.

The strength of this study is justified by the heterogeneity of the data, which allowed for an exploration according to blood indexes and diagnostic made on the ED. Moreover, significant associations were found for a non-specific cohort and raised for two specific groups of pathologies, which could enable focused research for the diseases under these groups of pathologies.

## 5. Conclusions

This study confirmed how anemia and inflammation are prevalent in emergency scenarios, especially for elderly patients with chronic conditions or comorbidities that endorse extra challenges on clinical outcomes. We verified a significant linear correlation between Hb and CRP on the entire cohort of patients admitted to the emergency department; this association was kept significant after age, gender, and pathology group adjustments. Our data provide support for the use of CRP and the CRP/Hb ratio as predictors of hospitalization following ER discharge.

## Figures and Tables

**Table 1:** Descriptive frequencies of the studied cohort.

Characteristics		Overall *n* = 125 (%)
Gender	Female	62 (49.6%)
	Male	63 (50.4%)
Age Group	<65	52 (41.6%)
	≥65	73 (58.4%)
Clinical condition	Anemia only	11 (8.8%)
	Inflammation only	31 (24.8%)
	Anemia and inflammation	45 (36.0%)
	No anemia either inflammation	38 (30.4%)
Diagnosis	I—Infectious diseases	12 (9.6%)
	II—Neoplasms (active cancer)	7 (5.6%)
	III—Hematological diseases	1 (0.8%)
	IV—Endocrine, nutritional, and metabolic disorders	1 (0.8%)
	V—Mental and behavioral disorders	6 (4.8%)
	VI—Diseases of the nervous system	7 (5.6%)
	VII—Diseases of the ear and mastoid process	3 (2.4%)
	IX—Diseases of the circulatory system	20 (16.0%)
	X—Diseases of the respiratory system	16 (12.8%)
	XI—Diseases of the digestive system	15 (12.0%)
	XII—Diseases of the skin and subcutaneous tissue	3 (2.4%)
	XIII—Diseases of the musculoskeletal system	6 (4.8%)
	XIV—Diseases of the genitourinary system	23 (18.4%)
	XIX—Injury and poisoning	2 (1.6%)
	XX—External causes of morbidity and mortality	3 (2.4%)
Discharge	Hospitalization	30 (24.0%)
	Primary health care	66 (52.8%)
	Home	29 (23.2%)
Length of stay	<6	66 (52.8%)
	≥6	59 (47.2%)

Data represent number and (frequency). Discharge includes exit categories of the emergency department and length of stay in hours.

**Table 2:** Distribution of clinical conditions (anemia only, inflammation only, both anemia and inflammation, and neither of these) across each group of the cohort.

Clinical Condition		Anemia Only	Inflammation Only	Anemia and Inflammation	No Anemia or Inflammation
Gender	Female	5 (8.1%)	19 (30.6%)	19 (30.6%)	19 (30.6%)
	Male	6 (9.5%)	12 (19.0%)	26 (41.3%)	19 (30.2%)
Age Group	<65	3 (5.8%)	17 (32.7%)	6 (11.5%)	26 (50.0%)
	≥65	8 (11.0%)	14 (19.2%)	39 (53.4%)	12 (16.4%)
Diagnosis	I	2 (16.7%)	5 (41.7%)	3 (25.0%)	2 (16.7%)
	II	2 (28.6%)	0 (0.0%)	5 (71.4%)	0 (0.0%)
	VI	0 (0.0%)	2 (28.6%)	2 (28.6%)	3 (42.9%)
	IX	1 (5.0%)	7 (35.0%)	5 (25.0%)	7 (35.0%)
	X	0 (0.0%)	3 (18.8%)	10 (62.5%)	3 (18.8%)
	XI	2 (13.3%)	3 (20.0%)	6 (40.0%)	4 (26.7%)
	XIV	3 (13.0%)	8 (34.8%)	7 (30.4%)	5 (18.8%)
Discharge	Hospitalization	1 (3.3%)	7 (23.3%)	20 (66.7%)	2 (6.7%)
	Primary health care	8 (12.1%)	19 (28.8%)	18 (27.3%)	21 (31.8%)
	Home	2 (6.9%)	5 (17.2%)	7 (24.1%)	15 (51.7%)
Length of stay	<6	6 (9.1%)	14 (21.2%)	20 (30.3%)	26 (39.4%)
	≥6	5 (8.5%)	17 (28.8%)	25 (42.4%)	12 (20.3%)

Data represent number and (frequency). Discharge includes exit categories of the emergency department and length of stay in hours.

**Table 3:** Correlation between Hb and CRP in the studied subgroups, according to main characteristics and clinical conditions.

Clinical Condition		Anemia Only	Inflammation Only	Anemia and Inflammation	No Anemia or Inflammation	All
Gender	Female	0.44 (0.450)	−0.26 (0.284)	−0.37 (0.112)	0.04 (0.867)	−0.37 (0.003)
	Male	−0.58 (0.222)	0.48 (0.108)	−0.26 (0.206)	−0.14 (0.567)	−0.56 (<0.001)
Age group	<65	0.50 (0.667)	−0.08 (0.736)	−0.03 (0.957)	−0.17 (0.402)	−0.30 (0.03)
	≥65	−0.36 (0.386)	0.01 (0.976)	−0.28 (0.087)	0.36 (0.252)	−0.43 (<0.001)
Diagnosis	I	-	−0.15 (0.805)	−0.50 (0.667)	-	0.28 (0.931)
	II	-	-	−0.46 (0.434)	-	−0.05 (0.908)
	VI	-	-	-	-	−0.82 (0.023)
	IX	-	0.05 (0.908)	−0.80 (0.104)	0.07 (0.878)	−0.28 (0.228)
	X	-	-	−0.69 (0.029)	-	−0.77 (0.001)
	XI	-	-	−0.37 (0.468)	−0.32 (0.684)	−0.51 (0.053)
	XIV	-	0.24 (0.570)	−0.18 (0.702)	0.56 (0.322)	−0.22 (0.325)
Discharge	Hospitalization	-	0.02 (0.969)	−0.45 (0.047)	-	−0.38 (0.038)
	Primary health care	−0.11 (0.797)	−0.08 (0.745)	−0.21 (0.413)	−0.08 (0.726)	−0.41 (0.001)
	Home	-	−0.70 (0.188)	−0.18 (0.699)	0.058 (0.837)	−0.47 (0.009)
Length of stay	<6	0.13 (0.802)	−0.34 (0.242)	−0.33 (0.156)	−0.12 (0.569)	−0.44 (<0.001)
	≥6	−0.80 (0.588)	0.08 (0.768)	−0.29 (0.154)	0.11 (0.741)	−0.48 (<0.001)
	All	−0.18 (0.588)	−0.10 (0.598)	−0.30 (0.044)	−0.05 (0.749)	−0.42 (<0.001)

Data represent the Spearman’s rank correlation coefficients (rs) with the respective level of significance (*p*).

**Table 4:** ROC curve results for blood parameters in the cohort.

Blood Parameters	Cut Off	AUC (95% CI)	*p*	Sensitivity	Specificity
CRP	67.55	0.811 (0.719–0.902)	<0.001	60.0%	87.4%
CRP/Hb	1.32	0.804 (0.711–0.898)	<0.001	80.0%	68.4%

AUC: area under the curve, CI: confidence interval.

## Data Availability

The data presented in this study are available on request to the corresponding author.
